# Radial growth resilience assessment and management implications for major planted forests in the west loess plateau based on improved parameters

**DOI:** 10.3389/fpls.2026.1946831

**Published:** 2026-09-23

**Authors:** Yucheng Xiao, Xiaomei Peng, Quanyan Tian, Cunwei Che, Jingrong Su

**Affiliations:** 1 Key Laboratory of Ecological Safety and Sustainable Development in Arid Lands, Northwest Institute of Eco-Environment and Resources, Chinese Academy of Sciences, Lanzhou, GanSu, China; 2 College of Geography and Environmental Science, Northwest Normal University, Lanzhou, GanSu, China; 3 Key Laboratory of Resource Environment and Sustainable Development of Oasis, Lanzhou, GanSu, China; 4 College of Surveying and Geoinformation, Lanzhou Resources & Environment Voc-Tech University, Lanzhou, GanSu, China

**Keywords:** arid Loess Plateau, growth resilience, improved parameters, plantation management, planted forest

## Abstract

Under the background of global climate change, frequent and severe drought have led to forest decline and tree mortality, ultimately threatening the structure, function, and stability of forest ecosystems. However, forest ecosystems and individual trees exhibit a certain degree of growth resilience to the impacts of drought. A profound understanding of the growth resilience of forest ecosystems and individual trees to drought is of great significance for maintaining the stability of forest ecosystems, particularly for plantation ecosystems. Based on an improved resilience assessment framework (maximum averaged growth reduction and recovery rate with the flexible time window), this study compared the radial growth resilience of major tree and shrub species between planted forests across different sub-climatic zones of the west Loess Plateau and corresponding natural forest species in adjacent area. The results indicate that: 1) Compared to plantations, natural forest tree species exhibit higher growth resilience. 2) *Platycladus orientalis*, *Hippophae rhamnoides*, and *Caragana korshinskii* possess high growth resilience, rendering them highly suitable for afforestation across the arid regions of the Loess Plateau. In contrast, *Picea crassifolia* and *Pinus tabulaeformis* showed lower resilience and should be confined to high altitude, semi-arid zones with higher precipitation. 3) In terms of plantation management in areas with insufficient precipitation, appropriate silvicultural interventions, such as rainwater harvesting, soil moisture conservation, and stand tending, must be supplemented to maintain the healthy and sustainable development of planted forests. These findings provide a scientific basis for species selection and adaptive plantation management on the Loess Plateau.

## 1 Introduction

Forest ecosystems play a vital role in terrestrial ecosystems. Against the backdrop of global climate change, frequent and severe drought not only increase water evapotranspiration demands in forest ecosystems but also lead to forest decline and tree mortality, ultimately affecting forest structure, function, and stability (Cui et al., 2025; Han et al., 2025a; Liang et al., 2019), particularly in regions where water availability is limited (Jiang et al., 2025). Therefore, gaining a deeper understanding of the mechanisms underlying the responses of forest ecosystems and individual trees to drought, as well as their growth resilience, is of great significance for maintaining the stability of forest ecosystems, especially for planted forest ecosystems.

China launched its largest-scale ecological restoration initiative, the Grain for Green Program, in the 1990s, focusing heavily on the Loess Plateau. Consequently, vegetation cover on the Loess Plateau has increased to approximately 65%, with forest cover reaching 24% by 2021 (Chen et al., 2024). Vegetation simulations indicate that the vegetation cover threshold for the Loess Plateau ranges between 53% and 65%. Global climate models project an increase in the frequency and intensity of extreme weather events as a result of anthropogenic climate change during this century (IPCC, 2013; Huang et al., 2018; Spinoni et al., 2019; Sun et al., 2019). Consequently, ecosystems in the Loess Plateau will face risks of decline and reduced stability in the future (Chen et al., 2024).

Assessing how forest ecosystems and individual trees have responded to past climatic events can clarify their potential adaptive capacity and growth resilience under future climate scenarios, thereby informing forest management policymaking (Yang et al., 2025; Li et al., 2025a). Introducing of the concepts of ecosystem resilience (Holling, 1973) and event point year (Schweingruber et al., 1990) relay the theoretical framework foundation for the resilience assessment of individuals, stands, populations, and ecosystems (Jetschke et al., 2019). The event point year was defined as the years in which a remarkable growth increase or reduction at the individual-tree level (like very narrow/wide ring widths, RW) or the stand level (lower/higher ring width index, RWI) (Van der Maaten-Theunissen et al., 2015; Jetschke et al., 2019). The identification of all event point years fundamentally relies on established threshold criteria, such as the requirement by Schröder (2013) that a certain quantile or statistical deviation of index values (e.g., mean −1SD or mean +1SD) falls below or exceeds a specified threshold at the stand level. Currently, the moving window normalization approach is widely applied to identify event point years. By evaluating the degree of growth deviation within local temporal windows while integrating responses at both the individual tree and population levels, this method effectively filters out background trends and precisely detects the relative abnormal growth responses of trees to extreme climatic events (Jetschke et al., 2019).

Lloret et al. (2011) decompose resilience into four components: resistance, recovery, resilience and relative resilience, and proposed an assessment method for tree growth resilience based on the low-growth episodes of basal area increment (BAI) and drought indices. Subsequently, numerous researchers have investigated the growth resilience of trees and forest ecosystems using parameters such as RW, RWI, BAI, tree-ring stable isotopes, and the normalized difference vegetation index (NDVI) (Merlin et al., 2015; Fang and Zhang, 2019; Fang et al., 2020; Che et al., 2023a, 2023b, 2025; Han et al., 2025b; Jiang et al., 2025; Li et al., 2025b; Ma et al., 2025). Based on a comprehensive assessment of different tree species, stand ages, drought indices, and tree growth parameters, Schwarz et al. (2020) suggested that it is more appropriate to assess plantation growth resilience using RWI. Because the RWI are measures derived after removing tree growth trends and primarily reflect the influence of climatic or environmental factors.

Studies on the climate response characteristics of multiple tree species in the arid zone of the Loess Plateau indicates that the radial growth of various natural and planted arbor and shrub species, including *Platycladus orientalis*, *Pinus tabulaeformis*, *Picea crassifolia*, *Caragana korshinskii* (a native desert shrub in the Alxa Desert, west of Loess Plateau, China), and *Hippophae rhamnoides* is significantly and positively correlated with growing-season precipitation and the standardized precipitation evapotranspiration index (SPEI). While, significantly and negatively correlated with high growing-season temperatures, indicating that growth is primarily constrained by low regional precipitation and the dominant arid climate (Che et al., 2022a, 2022b, 2023a, 2023b, 2025; Han, 2020; Su et al., 2024a; Xiao et al., 2024). In summary, the low-index episodes in these standard chronologies reflects the effects of atmosphere and soil drought, which include high stand density and evapotranspiration increasing with stand age (Che et al., 2022a, 2022b, 2023a, 2023b; Xiao et al., 2015, 2019).

Up to now, there remains a lack of comprehensive and in-depth understanding of the growth resilience of major afforestation tree species and their populations on the Loess Plateau to extreme climate events. This study applies the theory of tree growth resilience and, based on optimized and improved methods for identifying event point years and calculating parameters, assesses the growth resilience of several major planted tree species in the arid regions of the Loess Plateau, including the same species of the plantation and natural forest, the same plantation species in the different sub-climate zones and the different species in the same sub-climate zone. This study aims to provide scientific support for tree species selection and forest management decisions in the arid regions of Loess Plateau and similar environments.

## 2 Study area and data processing methods

### 2.1 Study area and materials

The study area is located in the western Loess Plateau and the nearby desert area (**Figure 1**). The region spans four distinct sub-climatic zones defined by annual precipitation: sub-humid (above 500 mm), semi-arid (around 400 mm), arid (200 to 300 mm), and arid desert (below 200 mm).

**Figure 1:**
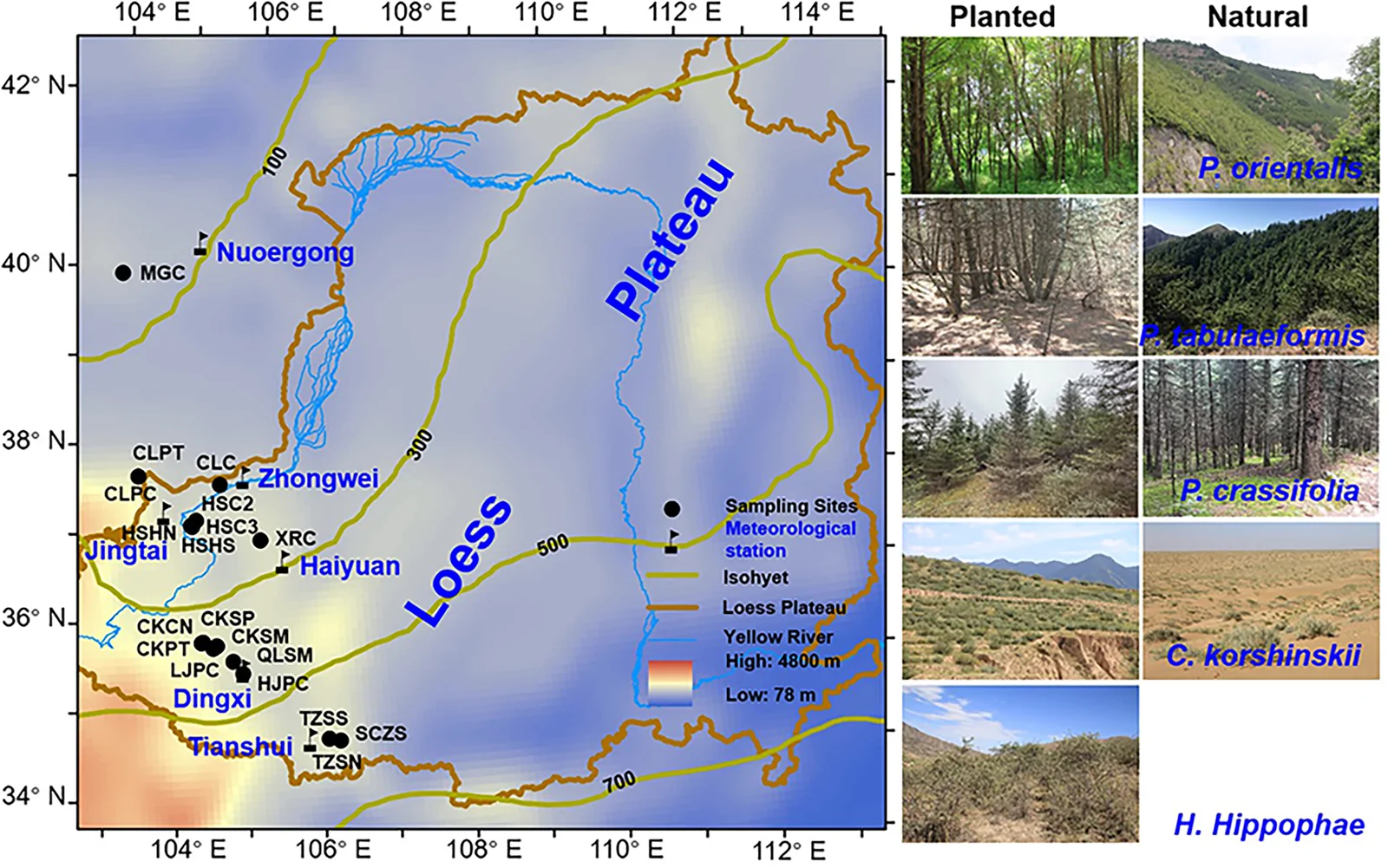
Sampling site distributions and plot landscape.

Sampling targeted both natural and planted forests of major tree and shrub species across this region: *P. orientalis*, *P. tabulaeformis*, *P. crassifolia*, *C. korshinskii*, and *H. rhamnoides.* Among these, *C. korshinskii* and *P. orientalis* occupy the largest afforestation areas on the Loess Plateau. Plantation structures include pure and mixed stands (often arranged in alternating rows or belts), established using site-preparation methods such as fish-scale pits, horizontal ditches, and level terraces. Most plantations have not undergone intensive silvicultural management like pruning or thinning.

Tree-ring samples from the aforementioned natural and planted forests of arbors and shrubs were collected sequentially from 2017 to 2023 (our team’s work). At each sampling site, more than 20 stem discs or more than 30 increment cores were obtained. Standard RW chronologies were established for each site in accordance with standard dendrochronological processing procedures and methods. The statistics characteristics of the habitat conditions, samples and RW chronologies for each site are detailed in **Table 1**. Because the chronologies of the planted forests are generally short, the starting year for the montane natural forests of *P. tabulaeformis* and *P. crassifolia* was fixed at 1955 to facilitate comparison over a similar time period, though their original chronology start years were 1848 and 1866 respectively. A part of these chronologies has been utilized in studies of climate reconstruction or radial growth resilience, such as the work on *P. tabulaeformis* and *P. crassifolia* by Han (2020) and Su (2024), and on *P. orientalis*, *C. korshinskii*, and *H. rhamnoides* by Che et al. (2022a; 2022b; 2023a; 2023b; 2025).

**Table 1:** Habitat and ring-width chronology information of arbor and shrub species at each sampling site.

Species	Origin	Sites	Location	Elevation (m)	Sub-climatic zone	Chronology length	Samples	MS	EPS	References
*Platycladus orientalis*	Natural	SCZS	106.266°E, 34.538°N	1151	Sub-humid	1972-2021	31/cores	0.326	0.900	Che et al., 2023b; 2025
	Planted	TZSS	106.117°E, 34.567°N	1181	Sub-humid	1955-2021	38/cores	0.220	0.905	
	Planted	TZSN	106.117°E, 34.567°N	1181	Sub-humid	1976-2021	35/cores	0.428	0.880	
	Planted	CKSP	104.467°E, 35.717°N	2037	Semi-arid	1968-2021	37/cores	0.355	0.919	
	Planted	CKSM	104.467°E, 35.717°N	2116	Semi-arid	1974-2021	40/cores	0.284	0.953	
	Planted	QLSM	104.673°E, 35.674°N	2245	Semi-arid	1980-2021	39/cores	0.256	0.973	
	Planted	DXS	104.620°E, 35.648N	2078	Semi-arid	1986-2020	37/discs	0.295	0.917	
*Pinus tabulaeformis*	Natural	CLPT	103.712°E, 37.611°N	2490	Arid mountain	1955-2018	50/cores	0.280	0.975	Han, 2020; Su, 2024
	Planted	CKPT	104.467°E, 35.717°N	2037	Semi-arid	1978-2020	37/cores	0.43	0.980	
	Planted	LJPT	104.883°E, 35.492°N	2185	Semi-arid	1987-2020	43/cores	0.30	0.980	
*Picea crassifolia*	Natural	CLPC	103.712°E, 37.611°N	2490	Arid mountain	1955-2018	48/cores	0.312	0.980	Han, 2020
	Planted	HJPC	105.006°E, 35.350°N	2320	Semi-arid	2004-2020	34/cores	0.21	0.990	Su, 2024
*Caragana korshinskii*	Natural	MGC	103.645°E, 39.889°N	1510	Arid desert	1979-2015	22/discs	0.395	0.962	Che et al., 2022b; 2023a; 2023b
	Planted	CLC	104.845E, 37.467°N	1630	Arid-sandy	2003-2020	27/discs	0.542	0.927	
	Planted	XRC	105.358°E, 36.821°N	1785	Arid-sandy	2004-2020	24/discs	0.195	0.913	
	Planted	HSC2	104.410°E, 37.031°N	1779	Arid	2005-2020	24/discs	0.197	0.908	
	Planted	HSC3	104.485°E, 37.085°N	1908	Arid	2008-2020	23/discs	0.308	0.915	
	Planted	QLMC	104.673°E, 35.674°N	2245	Semi-arid	1991-2021	22/discs	0.215	0.902	
	Planted	CKMC	104.620°E, 35.648°N	2078	Semi-arid	1977-2021	38/discs	0.157	0.905	
	Planted	CKCN	104.486°E, 35.724°N	2147	Semi-arid	1993-2020	28/discs	0.340	0.914	
	Planted	CKCS	104.486°E, 35.724°N	2116	Semi-arid	1990-2021	28/discs	0.208	0.877	
*Hippophae rhamnoides*	Planted	HSHN	104.410°E, 37.031°N	1358	Arid	2004-2020	22/discs	0.470	0.995	Che et al., 2022a
	Planted	HSHS	104.424°E, 37.019°N	2059	Arid	2005-2020	22/discs	0.365	0.995	

Samples include disc/cores. MS, mean sensitivity; EPS, expressed population signal.

### 2.2 Data sources and analytical methods

#### 2.2.1 Identification of event point years and time windows

This study adopts the low-index episodes of the RW chronologies to identify event point years (Schröder, 2013). Specifically, years in the RW chronology with an index lower than one standard deviation below the mean (*I* < mean - 1δ) are identified as event point years (**Figure 2A**). Due to the presence of drought legacy effects, when two or three consecutive low indices occurred, the minimum value among them is taken as the event point year (Ep).

**Figure 2:**
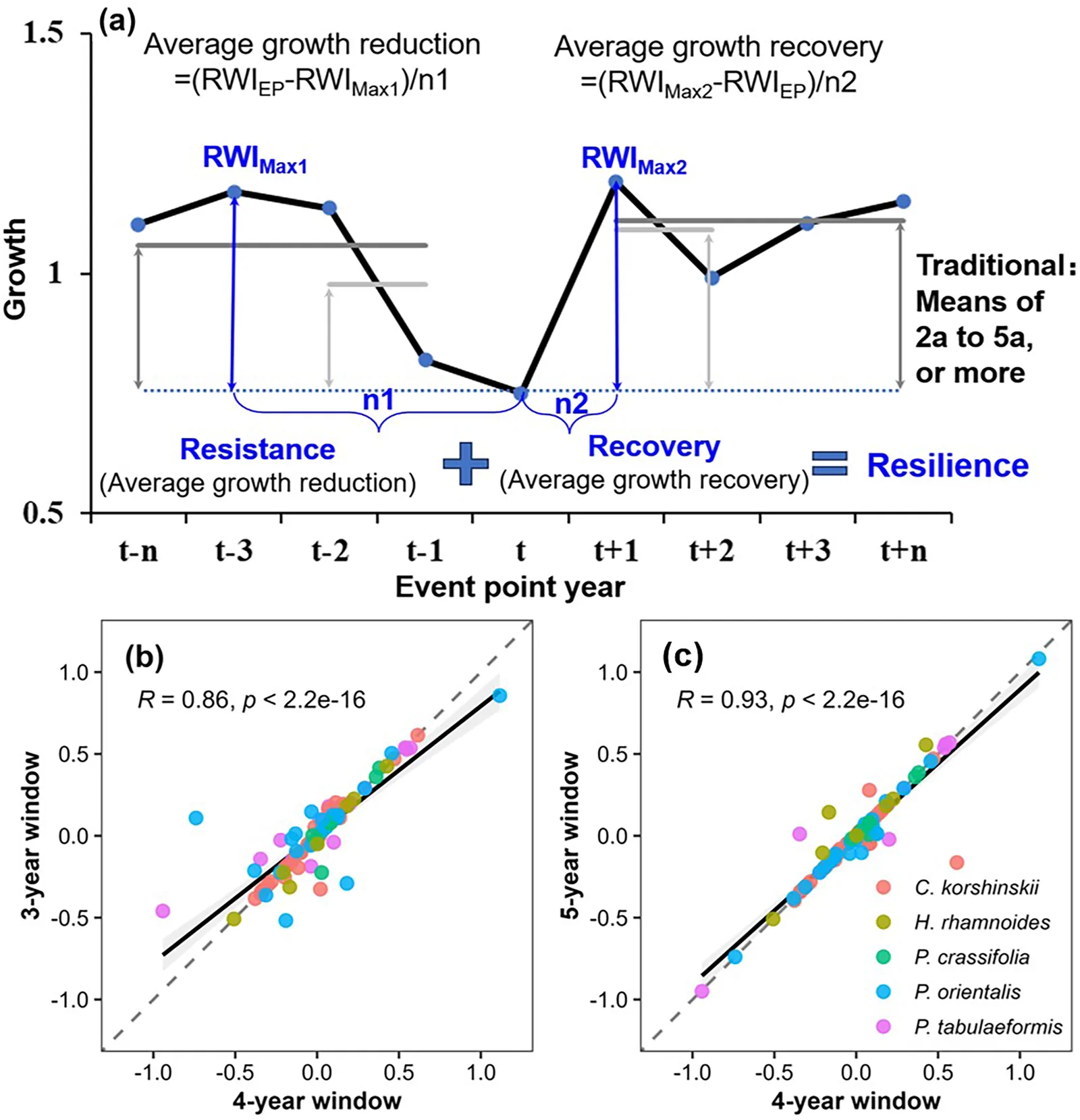
Calculation rationale and window selection of event point year. **(A)** Schematic of parameter and time window selection; **(B)**, **(C)** Sensitivity analysis of ARS calculated across different time windows. Calculations are based on the sampling sites investigated in this study (**Table 1**). Solid black lines and gray shaded areas denote linear regression fits and their 95% confidence intervals; dashed gray lines represent the 1:1 reference lines (y = x). Pearson correlation coefficients (R) and significance levels (*P*) between the two temporal windows are shown in the respective panels.

Research on climate change on the Loess Plateau from 1961 to 2010 indicated that the spring drought index in this region exhibited a significant high frequency oscillation cycle of 3–4 years (Yao et al., 2014). Simultaneously, temperature and precipitation variations in the semiarid zone of the Loess Plateau were predominantly characterized by cycles of 3, 7, and 10 years (Xie, 2016). Radial growth variations of major tree species in the surrounding mountains also displayed multiscale cyclic patterns, such as an approximate 4-year period (Han, 2020).

Sensitivity analyses using windows of 3, 4, and 5 years demonstrated that the consistency of average growth resilience (ARS) calculation results increased with expanding window length (**Figures 2B, C**). However, employing a window of 5-year or longer substantially increases the risk of compounding interference from subsequent drought events. In contrast, a 4-year window fully captures the entire trajectory from growth suppression to peak growth while effectively avoiding the confounding impacts of subsequent consecutive droughts.

Integrating the regional climate variation patterns of the Loess Plateau and adjacent areas with tree growth cycles, and taking into account the generally short chronological sequences of plantations and frequent drought occurrences, this study defines the pre-event reduction and post-event recovery window as the 4-year before and after the event point year.

#### 2.2.2 Definition and calculation of resistance, recovery and resilience

Considering the research conventions in growth resilience, this study adopts 4-year before and after the event as the assessment window, while combining resistance and recovery to calculate growth resilience (**Figure 2A**).

To calculate these indices, this study adapts the multi-year average reduction rate proposed by Schwarz et al. (2020) as the parameter to represent resistance, but expresses the reduction rate as a negative value. The average growth reduction (ARD), representing resistance, is calculated by subtracting the maximum RWI within the 4-year post-event from the RWI of the event point year, and then dividing by the time length between those two specific years (Equation 1):

(1) $$ARD=\frac{RWI_{Ep}-RWI_{Max1}}{n1}$$

The average growth recovery (ARC) is calculated by subtracting RWI of event point year from the maximum RWI within the 4-year post-event, and then dividing by the time length between those two years (Equation 2):

(2) $$ARC=\frac{RWI_{Max2}-RWI_{Ep}}{n2}$$

The average growth resilience (ARS) before and after the event point year is determined by the sum of the average growth reduction and the average growth recovery (Equation 3):

(3) ARS=ARD+ARC

In these equations, RWI_EP_ is the RWI of the event point year. RWI_Max1_ and RWI_Max2_ are the maximum RWI values within the 4-year pre- and post-event points, respectively. *n1* and *n2* are the length of period from the maximum RWI within the 4-year post-event point.

An ARS > 0 indicates that the ARC outpaces the ARD, signifying high population or ecosystem stability. Conversely, an ARS < 0 implies that ARD exceeds ARC, indicating vulnerability. By integrating these metrics into a single coordinate system, it enables direct interspecific and inter-site comparisons.

To overcome the limitations of short plantation chronologies and sample sizes per site, data from adjacent plantation sites of the same species were pooled for regional analysis. Box plots were used for visualization, and independent t-tests were applied to evaluate statistical significance.

## 3 Results and analysis

### 3.1 Comparison of radial growth resilience of same forest species among the different sub-climatic zones

#### 3.1.1 P. orientalis

Natural *P. orientalis* forests generally exhibited higher ARD, ARC, and ARS values than planted forests, though these differences were not statistically significant (**Figure 3A**). The ARD of planted *P. orientalis* was significantly higher in the sub-humid zone than in the semi-arid zone (*P* < 0.05). However, its ARC and ARS values were lower in the sub-humid zone. This indicates that while *P. orientalis* plantations in semi-arid zones are more vulnerable to initial drought stress, their strong post-event recovery capacity yields higher overall growth resilience when conditions improve. Crucially, the ARS values for both natural and planted stands remained positive, indicating that the ARC exceeds the initial ARD and that drought exerts a relatively mild long-term constraint on this species on the Loess Plateau.

**Figure 3:**
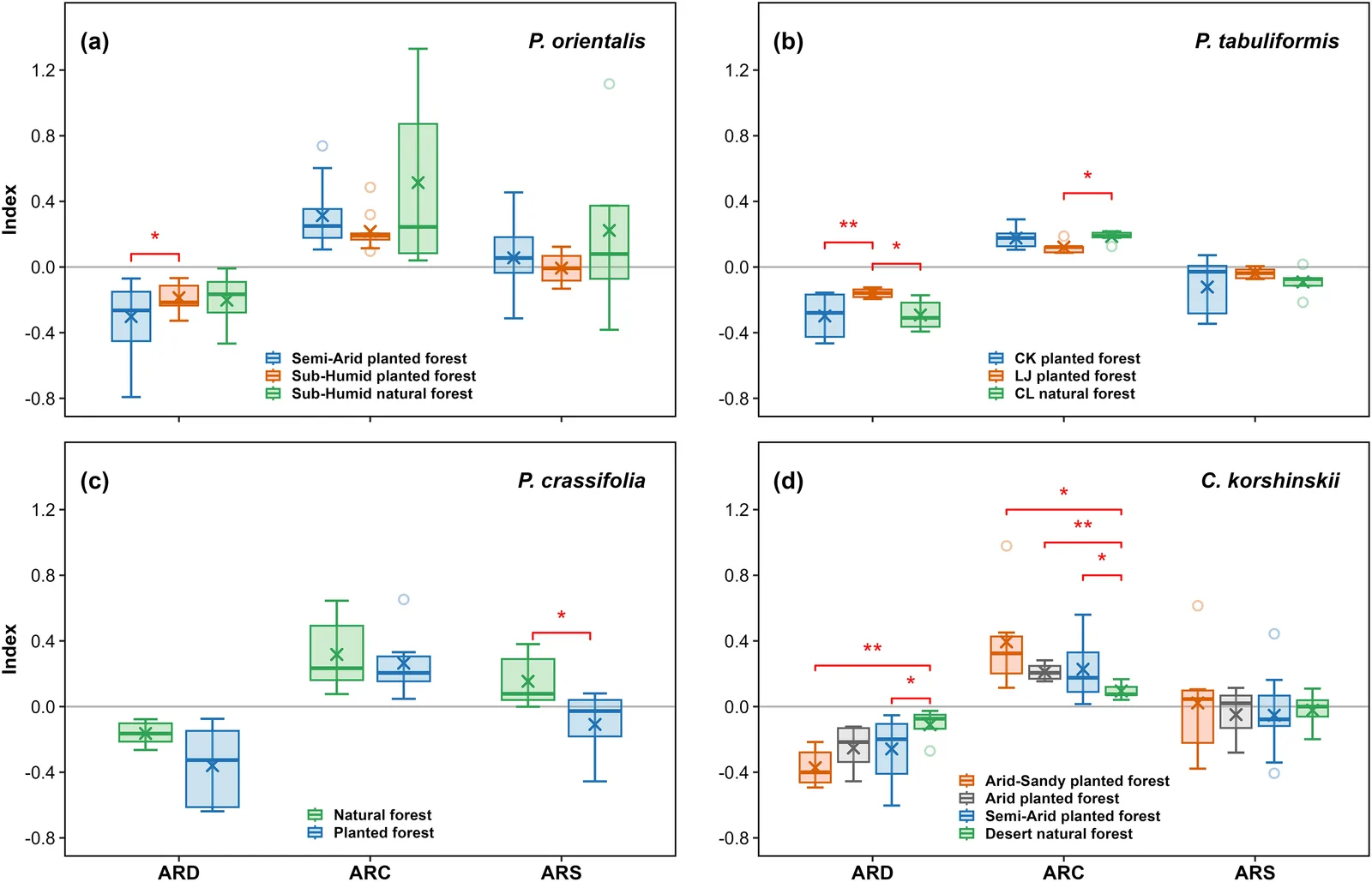
Box plots of radial growth resilience for different tree species in natural and planted forests. **(A)** *P. orientalis*; **(B)** *P. tabulaeformis*; **(C)** *P. crassifolia*; **(D)** *C. korshinskii.* Asterisks indicate statistically significant differences: *P < 0.05, and **P < 0.01.

#### 3.1.2 P. tabulaeformis

For planted *P. tabulaeformis*, the semi-arid LJPT site showed a significantly higher ARD than the semi-arid CKPT and natural montane CLPT sites (*P* < 0.05; **Figure 3B**). Conversely, the CKPT site displayed the highest ARC and ARS values, with its ARC being significantly higher than that of the LJPT site (*P* < 0.05). The natural CLPT site exhibited the lowest ARS. Notably, the ARS values for both natural and planted *P. tabulaeformis* were uniformly negative across all sites.

#### 3.1.3 P. crassifolia

The natural *P. crassifolia* forest (CLPC site) exhibited higher ARD, ARC, and ARS values than the semi-arid plantation site (HJPC site), with the difference in ARS reaching statistical significance (*P* < 0.05; **Figure 3C**). The ARS of the planted HJPC forest was negative, demonstrating that under rain-fed conditions, post-event recovery is slow and stands require multiple years to return to pre-growth levels.

#### 3.1.4 C. korshinskii

Natural and planted *C. korshinskii* exhibited complex variations across climate zones (**Figure 3D**). For ARD, the desert natural forest showed higher resistance than all plantation sites, differing highly significantly from the arid-sandy plantation (*P* < 0.01) and significantly from the sub-humid plantation (*P* < 0.05). For ARC, the arid-sandy and semi-arid plantations showed significantly higher recovery than the desert natural forest (*P* < 0.05 and *P* < 0.01, respectively). For ARS, values decreased in the following order: arid-sandy plantation > semi-arid plantation > desert natural forest > arid plantation, while inter-site differences were not up to the significant level. Except for the semi-arid plantation, ARS values were consistently positive across sites, indicating robust recovery potential in arid and sandy settings once drought stress abated.

### 3.2 Comparison of radial growth resilience among different planted tree species within the same sub-climatic region

#### 3.2.1 Semi-arid zone

In the semi-arid zone, ARD values decreased in the order of *P. tabulaeformis* > *C. korshinskii* > *P. orientalis* > *P. crassifolia*, but the interspecific differences were not up to the significant level (**Figure 4**). However, ARC followed the sequence of *P. orientalis* > *P. crassifolia* > *C. korshinskii* > *P. tabulaeformis*, with *P. orientalis* showing a significantly higher recovery capacity than *P. tabulaeformis* (*P* < 0.05). ARS values followed the same order as ARC, with *P. orientalis* outperforming *P. tabulaeformis* significantly (*P* < 0.05). Within this climate zone, ARS was uniformly negative for *P. crassifolia*, *P. tabulaeformis*, and *C. korshinskii*, but remained positive for *P. orientalis*. This confirms the superior eco-physiological adaptability of *P. orientalis* to the semi-arid climate on the Loess Plateau.

**Figure 4:**
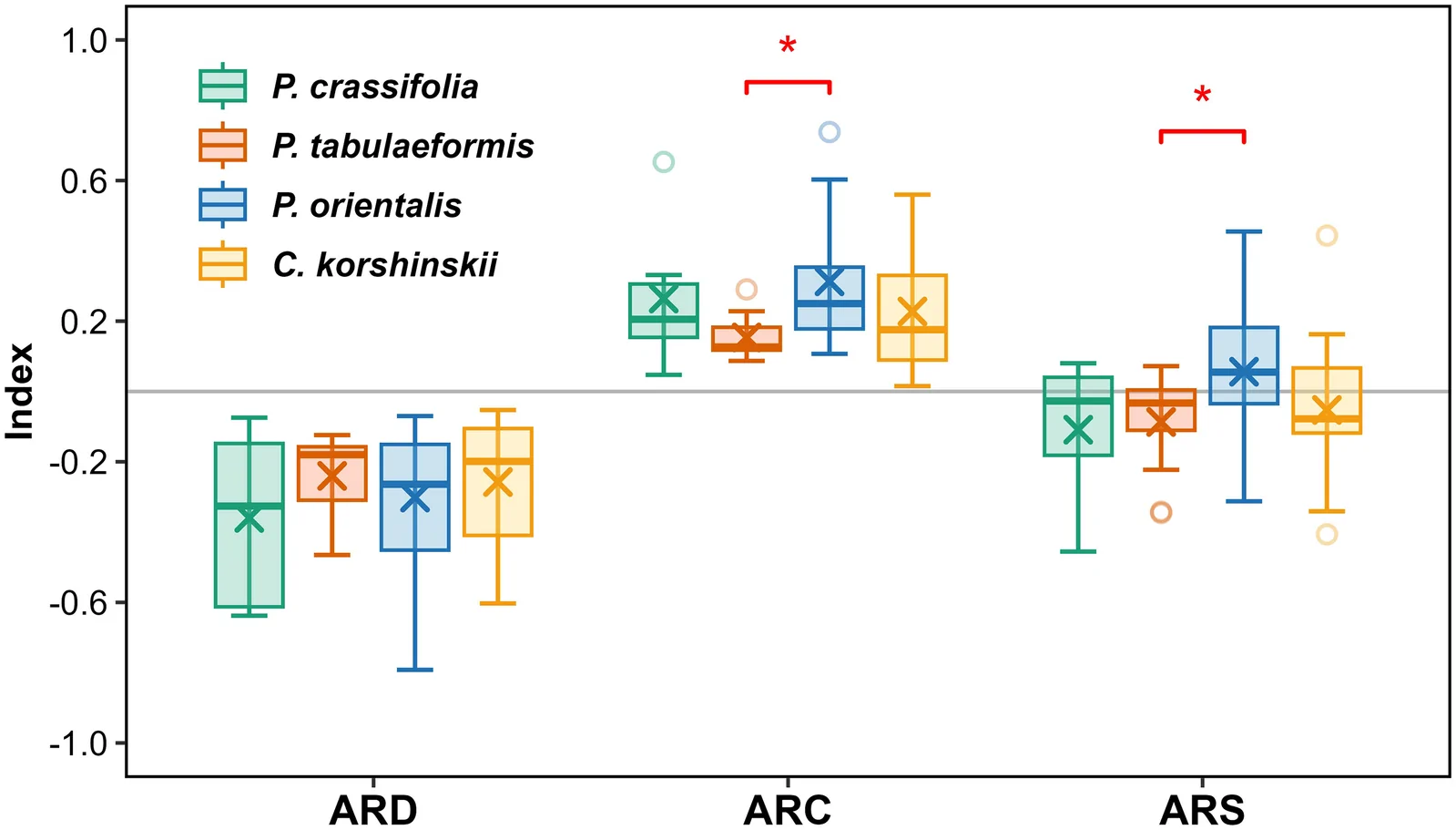
Box plots comparing the radial growth resilience of different planted tree species in the semi-arid zone. Asterisks indicate statistically significant differences: *P < 0.05, and **P < 0.01.

#### 3.2.2 Arid zone

No significant differences were detected across the three indices for the two shrub species in the arid zone (**Figure 5**). Planted *H. rhamnoides* had a lower ARD than *C. korshinskii*, indicating greater sensitivity to initial drought stress. However, *H. rhamnoides* compensated with higher ARC and ARS values. Both shrub species displayed positive ARS values, confirming that their ARC exceeded reduction rates, establishing them both as highly resilient options for regional ecological restoration.

**Figure 5:**
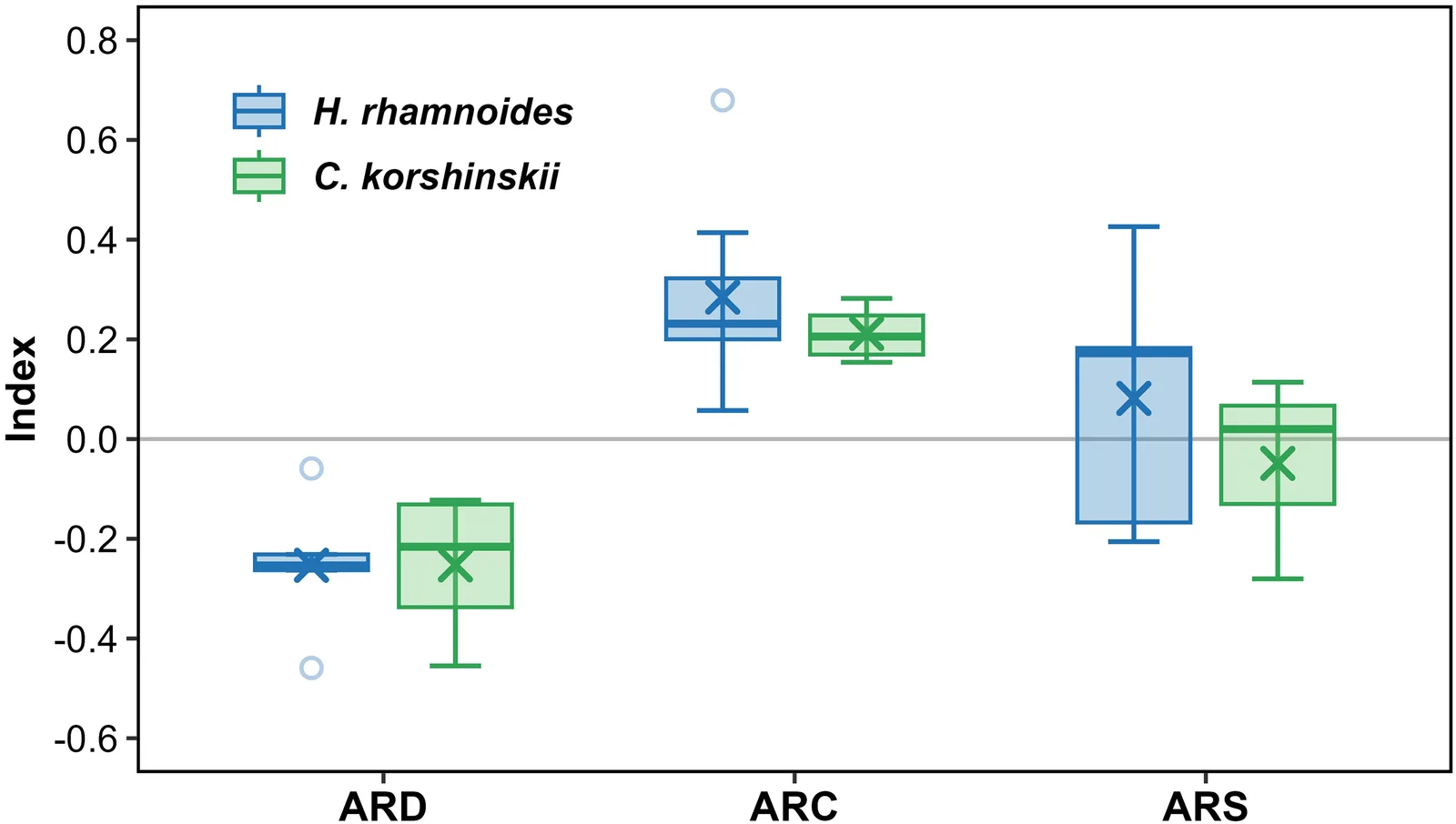
Box plots of growth resilience for *H. rhamnoides* and *C. korshinskii* plantations in the arid zone of the Loess Plateau. Asterisks indicate statistically significant differences: *P < 0.05, and **P < 0.01.

## 4 Discussion and conclusions

### 4.1 Parameter selection for growth resilience calculations

In previous studies, resistance and recovery were conventionally defined as the ratio of RWI during the event point year (RWI_EP_) to the multi-year average preceding it, and the ratio of the multi-year average following it to the same event value, respectively. Resilience was calculated as the ratio of the multi-year RWI average after and before event point year (Lloret et al., 2011; Fang and Zhang, 2019; Rahman et al., 2019; Fang et al., 2020; Che et al., 2023a, 2023b, 2025; Jiang et al., 2025; Cui et al., 2025). In these formulas, RWI_Ep_ serves simultaneously as the numerator of resistance and the denominator of recovery (**Figures 6A, B**). Consequently, the highly significant, strong negative correlation between the two may lead to distortions in the resilience calculation.

**Figure 6:**
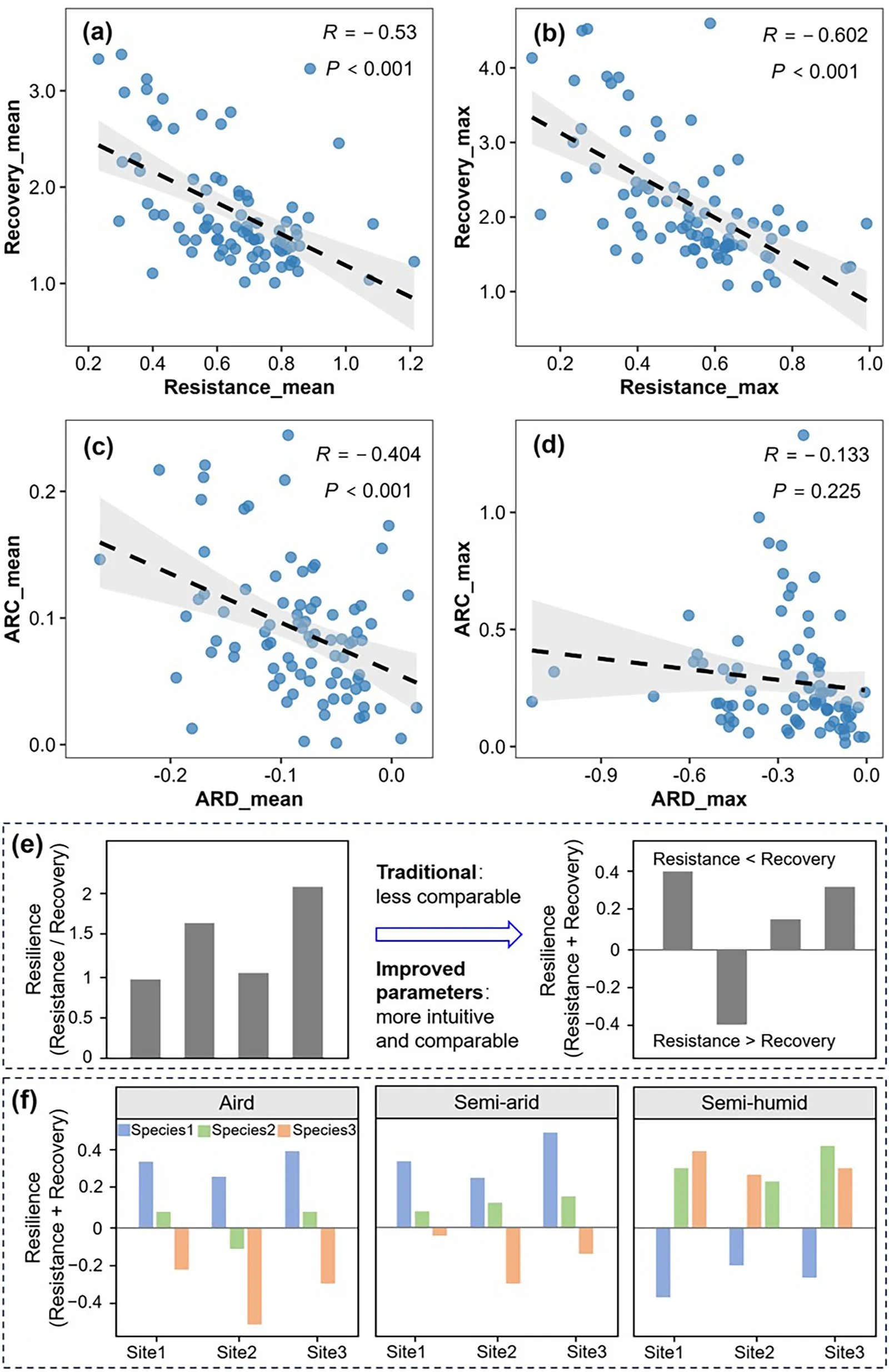
Schematic of the growth resilience based on improved parameters. **(A)** Traditional mean-RWI method (Lloret et al., 2011); **(B)** Traditional method with maximum-RWI; **(C)** The calculation method proposed in this study but using mean-RWI (Schwarz et al., 2020); and **(D)** The calculation method proposed in this study. Calculations are based on the sampling sites investigated in this study (**Table 1**). Dashed black lines and gray shaded areas denote the linear regression fits and their 95% confidence intervals, respectively. Pearson correlation coefficients (*R*) and significance levels (*P*) are annotated in each panel. **(E, F)** Intuitive advantages of the method proposed in this study.

Schwarz et al. (2020) proposed using the long-term ARD as a parameter for resistance, and defined the long-term ARC as the ratio of 1 minus resistance to the recovery time. The standardized RWI fluctuates between 0 and 2, with 1 representing the median of the RWI series. This definition treats the average RWI value of 1 as the expected value for recovery, representing only the potential ARC or probability that tree growth will reach the expected means (Che et al., 2023b, 2025), while ignoring the actual situation where tree growth recovers to levels above the long-term average. Moreover, the use of mean RWI values in the calculation smooths out fluctuations in radial growth, making it difficult to capture the abrupt declines induced by climatic anomalies, which ultimately overestimates ARD and underestimates ARC (**Figure 6C**). Therefore, whether based on the long-term average of the RWI prior to the event point year or on the selection of the expected value, both approaches underestimate the ARD, ARC, and ARS to some extent.

Based on tree-ring stable isotopes, Fang et al. (2020) investigated the growth resilience of juniper and fir trees on the Tibetan Plateau, showed that the legacy effects of event typically persist for 0.8 to 2.4 years, while the subsequent growth recovery period spans 1.3 to 3.0 years. Incorporating both these drought legacy effects and the primary cycles of regional arid climate variations, the present study identifies event point years as those in the RW chronology where the index falls below one standard deviation from the mean ( I<mean−1δ). Although the assessment window spans four years before and after the event, identifying the maximum RWI and its specific year within this window allows the study to capture the potential values of ARD and ARC more accurately. In the two aspects of ARS, the ARD is expressed as a negative value, maintaining consistency with the rising and falling trends of the ARC, while preventing the resilience calculation from distorting when RWI_Ep_ is shared as both the numerator and denominator in the formula.

Compared to the ratio of resistance to recovery (Lloret et al., 2011), the sum of these two values exhibits both positive and negative characteristics with ARS = 0 serving as a distinct baseline (**Figure 6E**), allowing for a more intuitive representation of growth resilience and facilitating comparisons among different tree species and sampling sites within the study area (**Figure 6F**).

### 4.2 Radial growth resilience across different species

The tree growth resilience is primarily influenced by a combination of factors, including tree species, climate, habitat, and stand structure (such as individual size, competition) (Li et al., 2025a). In the case of planted forests, in addition to the factors mentioned above, growth resilience is also influenced by planting density, mixed-species planting methods, stand age, and management practices (such as water harvesting methods in arid regions and silvicultural measures). Since management methods, site conditions, and mixed conditions vary among plantation species, while sample sites have consistent and relatively short forest ages, this section only compares the growth resilience of different species and their natural and plantation forests.

#### 4.2.1 P. orientalis

Our results indicate that the natural forest of *P. orientalis* in the sub-humid zone exhibited higher values across all three growth resilience indices than those of plantation sites spanning sub-humid to arid sub-climatic zones. Notably, the ARS for both natural and planted forests of *P. orientalis* is positive across different sub-climatic zones (**Figure 3A**). Studies at the daily, seasonal, and interannual scales show that the radial growth of *P. orientalis* is primarily influenced by soil moisture conditions and growing-season precipitation (Jiang et al., 2015; Xiao et al., 2019; Che et al., 2025) and is positively correlated with growing season precipitation and the drought index (SPEI), exhibiting heterogeneous characteristics across different sub-climatic zones (Che et al., 2025).

#### 4.2.2 P. tabulaeformis

The natural *P. tabulaeformis* forest (CLPT site) described in this paper is located at the western boundary of its natural range and is primarily subject to arid climatic stress (Han, 2020; Xiao et al., 2024; Su et al., 2024b). However, because its elevation is higher than that of the arid regions of the Loess Plateau, its ARS is greater than that of planted forests (**Figure 3B**). For the planted forests (CJPT and LJPT sites) located in the southern Loess Plateau, although regional precipitation has increased, the elevation of the afforestation areas has decreased and temperatures have risen. This has ultimately led to increased evapotranspiration in the forest stands, resulting in continued severe constraints from arid climatic conditions.

Coupled with the global trend toward warmer and drier climates, both natural and planted *P. tabulaeformis* forests exhibit negative ARS (**Figure 3B**). A meta-analysis of climate responses in the distribution range of *P. tabulaeformis* indicates that in areas with annual precipitation <400 mm, the primary factors limiting radial growth are growing season precipitation and drought (Su et al., 2024b). Furthermore, at the CKPT site, artificial managements were implemented around 2010, involving the retention of a single main stem per planting hole and the removal of lateral branches below 2 m, along with the use of horizontal ditches for rainwater collection and water conservation. These measures reduced the stand’s evapotranspiration to some extent. In contrast, the LJPT site received no silvicultural treatments and was established using a wide-water-platform afforestation method. Consequently, after the drought subsided, water consumption at the LJPT site remained higher than at the CKPT site, indicating lower ARC (**Figure 3B**). This demonstrates that tending measures play a crucial role in the recovery of planted forests, multiple studies have shown that intraspecific competition within stands can reduce the impact of events on to ARD and ARS (Jiang et al., 2025; Cui et al., 2025). The uniformly negative ARS values for both natural and planted forests of *P. tabulaeformis* indicate that this species is profoundly susceptible to regional drought stress on the Loess Plateau, a finding supported by a study in the Funiu Mountains within the temperate to sub-tropical transition zone (Li et al., 2025b).

#### 4.2.3 P. crassifolia

*P. crassifolia* is an endemic tree species found along the northeastern edge of the Qinghai-Tibet Plateau and extending into the central mountainous regions of the Loess Plateau. It is a moisture-loving, shade-tolerant species, primarily distributed on shaded and partially shaded slopes in high-altitude mountainous areas at elevations of 2,200–3,200 m, where the average annual temperature ranges from around 0 °C to 7–8 °C and the average annual precipitation ranges from approximately 300 mm to 700 mm. A meta-analysis indicates that across the entire distribution range of *P. crassifolia* precipitation and drought stress remain the primary factors influencing its radial growth, compared to temperature. Furthermore, the effects are most pronounced in areas with low precipitation and high temperatures (<200 mm and >7 °C) as well as in areas with low temperatures and high precipitation (>400 mm and <0 °C) (Su, 2024).

As the dominant tree species in mountain forests on the Loess Plateau, natural forests (CLPC site) of *P. crassifolia* exhibits the higher ARD, ARC and ARS compared to planted forests (HJPC site) in semi-arid regions (**Figure 3C**). Planted forests are located at lower elevations than natural forests and are more severely suppressed by low precipitation and high temperatures. Consequently, planted forests exhibit lower ARS, meaning that the ARC following events is slower than the ARD (**Figure 3C**).

#### 4.2.4 C. korshinskii

*C. korshinskii* is a drought-tolerant shrub capable of growing in areas with annual precipitation ranging from 100 mm to 550 mm. Naturally distributed in desert areas due to its well-developed root system and high drought tolerance, it has been widely introduced to the Loess Plateau (Xiao et al., 2015). Therefore, natural desert forests of *C. korshinskii* exhibit greater ARS than plantations in the various sub-climatic zones of the Loess Plateau.

However, as precipitation increases, *C. korshinskii* plantations demonstrate greater ARC than natural forests (**Figure 3D**). Concurrently, planted forests in arid sandy regions maintain high ARS due to the higher water permeability of sandy soils compared to loess parent material. In contrast, the relatively high precipitation in the semi-arid region of the Loess Plateau partially meets the water requirements for the growth of *C. korshinskii* plantations, resulting in taller shrubs than those found in natural desert *C. korshinskii* forests. Consequently, these plantations exhibit lower ARS and ARC when competing with herbaceous plants within the shrub canopy and the broader community (**Figure 3D**). Che et al. (2022b) compared the characteristics of the radial growth of *C. korshinskii* under different precipitation gradients and found that the limitation of drought on the growth of *C. korshinskii* gradually decreased with increasing precipitation.

With the exception of *C. korshinskii* plantations in the semi-arid region of the Loess Plateau, the ARS of *C. korshinskii* plantations in sandy and arid regions was positive, indicating that they can recover to normal growth levels after being affected by drought. In contrast, the tall shrub communities formed by relatively high precipitation in the semi-arid regions of the Loess Plateau result in higher soil water demand and water loss through evapotranspiration, to the extent that they are unable to recover to normal growth levels even after the drought subsides (Xiao et al., 2015; Che et al., 2023a).

### 4.3 Implications for afforestation and artificial management on the arid zone

Due to the impacts of climate change and historical human activities, natural coniferous tree species in the Loess Plateau region, such as *P. crassifolia*, *P. tabulaeformis*, and *P. orientalis*, are primarily confined to high-altitude mountainous areas characterized by relatively high precipitation and lower temperatures. Meanwhile, *C. korshinskii* serves as a primary dominant shrub species in arid desert zones, and *H. rhamnoides* is a widely distributed shrub species across the Loess Plateau. In contrast, the afforestation sites for the aforementioned coniferous tree species are often located at lower elevations than natural forests or in drier areas with lower precipitation. Furthermore, as these are even-aged forests, both increased stand transpiration and competition among individuals reaching a certain age will affect the tree growth resilience. For the desert shrub *C. korshinskii*, the transition of its habitat from shifting sand dunes in arid zones to the loess substrates of the Loess Plateau introduces vast discrepancies in soil water infiltration and water-holding capacities, ultimately leading to significant variations in growth resilience under drought influences.

The comparative results across different climatic zones and tree species on the Loess Plateau indicate that *P. orientalis* should be the species of choice for afforestation from semi-arid to sub-humid zones (**Figure 4**). However, when establishing *P. orientalis* plantation within extremely arid zones, supplementary artificial irrigation is required to guarantee the stable maintenance of these planted forests. Studies on the growth resilience of species in the Pinaceae and Cupressaceae trees indicate that Cupressaceae exhibit a higher capacity to adapt to compound events (Zhang et al., 2025). Concurrently, periodic thinning of planted forests can adjust stand structure, effectively reducing growth decline caused by drought and enhancing tree growth resilience (Jones et al., 2019).

For the native species *H. rhamnoides* and the desert shrub *C. korshinskii*, both exhibit good growth resilience and can be considered the preferred choices for afforestation shrubs (**Figure 5**). In afforestation practice, the *H. rhamnoides* should be planted in semi-arid areas on shaded slopes with favorable moisture conditions, and rainwater harvesting and water conservation measures should be implemented to reduce drought stress and enhance growth resilience. In the sub-humid regions of the Loess Plateau, *H. rhamnoides* and *C. korshinskii* plantations should timely coppice at appropriate age classes to maintain soil water balance and ensure the healthy and sustainable development of the forests (Xiao et al., 2015; Che et al., 2023a).

Studies on the photosynthetic characteristics of *P. orientalis*, *P. crassifolia*, and *P. tabulaeformis* on the Loess Plateau revealed that the diurnal variation in net photosynthetic rate (*P_n_*) of these three species undergoes a seasonal transition between unimodal and bimodal patterns (Li, 2005). Specifically, the diurnal *P_n_* curves exhibit a unimodal pattern during the early and late growing seasons, but shift to a bimodal pattern during the peak growing season (May to August), which is primarily driven by high-temperature and drought stress alongside stomatal conductance regulation (Cao, 2010). Given that the growth resilience of *P. crassifolia* and *P. tabulaeformis* plantations is severely suppressed by drought due to factors such as decreasing elevation, increased water loss through evapotranspiration, and competition among individuals within the even-aged forests (Huang et al., 2025), it is recommended that afforestation sites be primarily selected in semi-arid regions and areas with higher precipitation, as well as on shaded or semi-shaded slopes.

Previous research has shown that *H. rhamnoides* leaves possess thick cuticles, well-developed palisade tissue cells, and dense trichomes on the leaf surface, conferring high drought adaptability (Li et al., 2003). Meanwhile, the transpiration water consumption of *C. korshinskii* drops around midday (showing a bimodal pattern), whereas that of *H. rhamnoides* exhibits a unimodal pattern (Shen, 2005). Both shrub species have well-developed root systems with superior capacity for soil water absorption and utilization. This suggests that, compared with *H. rhamnoides*, the desert zonal shrub *C. korshinskii* possesses a stronger regulatory capacity under drought stress. Additionally, initial planting density should be controlled, appropriate rainwater harvesting and water conservation measures should be implemented, and age-appropriate stand tending should be carried out to meet the water requirements.

### 4.4 Conclusions and future prospects

Based on the current study regarding parameter selection for tree radial growth resilience assessments, this study proposes using the maximum RWI and its corresponding timing within a four-year window before and after event as the optimal parameters. This assessment framework can more accurately and objectively reflect growth resilience across different regions and tree species.

By applying this newly developed assessment framework, this study compared the radial growth resilience of various arbor and shrub species across natural and planted forests within different sub-climatic zones of the Loess Plateau. The findings indicate that *P. orientalis*, *H. rhamnoides*, and *C. korshinskii* are highly suitable for afforestation throughout the entire Loess Plateau region. In contrast, *P. crassifolia* and *P. tabulaeformis* should be restricted to the semi-arid zones and areas characterized by higher precipitation on the Loess Plateau. Furthermore, in areas with insufficient precipitation, appropriate artificial management practices must be supplemented to maintain the healthy and sustainable development of these planted forests.

Given that the large-scale plantations established under the Grain for Green Program are relatively young, long-term resilience data remain limited. This study only focused on five major species. Future research should implement comparative investigations encompassing a broader range of tree species, diverse soil-site conditions, and varying management practices (such as mixing planting and thinning intensities) to achieve a comprehensive understanding of forest stability and population dynamics on the Loess Plateau.

## Data Availability

The raw data supporting the conclusions of this article will be made available by the authors, without undue reservation.
